# BIBF1120 Protects against Diabetic Retinopathy through Neovascularization-Related Molecules and the MAPK Signaling Pathway

**DOI:** 10.1155/2023/7355039

**Published:** 2023-04-28

**Authors:** Xin Cao, Tao Li, Yongshen Tian, Yajing Tian, Chuang Gao, Dongmei Zhang, Yu Song

**Affiliations:** Department of Ophthalmology, Affiliated Hospital 2 of Nantong University, Nantong, Jiangsu 226000, China

## Abstract

Diabetic retinopathy (DR) is one of the microvascular complications of diabetes mellitus and a major pathological feature of neovascular DR. These patients potentially experience vision impairment and blindness. Platelet-derived growth factor receptor *β* (PDGFR*β*), fibroblast growth factor receptor 1 (FGFR1), and vascular endothelial growth factor receptor 2 (VEGFR2) are implicated in the DR pathogenesis. Nintedanib (BIBF1120) is an oral selective dual receptor tyrosine kinase (RTK) inhibitor of VEGFR2, FGFR1, and PDGFR*β*. In this study, intravitreal injection of BIBF1120 blocked the phosphorylation of VEGFR2, FGFR1, PDGFR*β*, and MAPK signaling pathway proteins in a streptozotocin (STZ)-induced diabetic retinopathy mouse model. In *in vitro* cell experiments, BIBF1120 did not change cellular activity under normal conditions, while it further suppressed the tube formation, migration, and proliferation of high glucose-induced human retinal microvascular endothelial cells (HRMECs). Additionally, BIBF1120 blocked the phosphorylation of p38, JNK, and ERK1/2 in high glucose-treating HRMECs. Our results indicate that the BIBF1120 treatment can be a novel potential drug to protect against DR.

## 1. Introduction

Diabetic retinopathy (DR) is one of the prevalent microvascular complications of diabetes mellitus and the main cause of blindness in adults aged 20–74 years old [1]. DR is classified into nonproliferative DR (NPDR) in the early stage and proliferative DR (PDR) in the later stage. Retinal neovascularization is the main pathological feature of PDR, which develops as a result of the retinal microvascular system damage, including pericyte loss, optic neural retinal abnormality, and neovascularization [2]. These pathological features can lead to serious complications, including diabetic macular edema and retinal neovascularization, resulting in visual impairment and blindness in DR patients [3]. Retinal capillary endothelial cell proliferation is a prerequisite for neovascularization.

Vascular endothelial growth factor (VEGF), a key factor in neovascularization-related intraocular diseases, binds to endothelial cell surface receptors through three vascular endothelial growth factor receptor (VEGFR) variants (VEGFR-1, VEGFR-2, and VEGFR1-3), despite the pathophysiology of ocular pathological neovascularization being complicated. Among them, VEGFR2 which is primarily expressed in endothelial cells, mediates pathological angiogenesis [4]. Studies have shown that inhibiting VEGFR2 downstream signaling interferes with angiogenesis [5]. Intravitreal injection of anti-VEGF, which can prevent abnormal angiogenesis and reduce exudation, has been used to address such diseases [6]. Anti-VEGF drugs have achieved remarkable success, but there are still many disadvantages such as nonreactivity and drug resistance in some patients [7]. These problems might be due to other angiogenesis compensation mechanism, including the fibroblast growth factor (FGF), the receptor (FGFR), the platelet-derived growth factor (PDGF), and the receptor (PDGFR) pathway, which can also promote the growth of neovascularization, so a new generation of more targeted antiangiogenic agents is needed.

FGFR-1, mainly expressed in endothelial cells, induces cell proliferation, survival, differentiation, and angiogenesis through multiple signal cascades, including activation signal transduction and transcriptional activators [8, 9]. PDGFR*β*, a member of the PDGFR family of receptor tyrosine kinases (RTKs), transduces angiogenesis and proliferation of PDGF and is more strongly expressed in retinal tissue under pathological conditions. In addition, PDGF and its receptor-mediated pathways play a critical part in vascular maturation [10, 11]. Therefore, due to the key role of the FGFR-1 and PDGFR signaling pathways in the pathogenesis of several blinding neovascular diseases of the eye, efforts have been focused on the development of inhibitors targeting the tyrosine kinase pathway.

Additionally, mitogen-activated protein kinase (MAPK) signaling pathways can trigger cell apoptosis, proliferation, mitosis, and gene transcription in response to high glucose stress [12, 13]. The survival or programmed cell death of human microvascular endothelial cells is strongly influenced by MAPK pathway activation [14]. In this family, stress-activated protein kinase 2 (p38), C-Jun NH2-terminal kinase (JNK), and extracellular signal-regulated kinase (ERK) pathways are associated with signal transduction in angiogenesis [15–17]. MAPK family members are phosphorylated by cascade regulation and activated by extracellular stimulation [18], and studies have shown that this pathway assists in developing DR [19, 20]. Therefore, we groped the potential mechanism of the MAPK signaling pathway in the formation of neovascularization in DR.

BIBF1120 (nintedanib) is an oral selective receptor tyrosine kinase inhibitor targeting a variety of growth factor receptors (VEGFRs, FGFRs, and PDGFRs) that has antiangiogenic effects and has been proven to treat various kinds of cancer, including gynecologic malignant tumors, idiopathic pulmonary fibrosis, prostate cancer, colorectal cancer, renal cell carcinoma, and hepatocellular carcinoma [21]. BIBF1120 also inhibits the MAPK signaling pathway to reduce the density of blood vessels, vascular permeability and tumor growth [22, 23]. Therefore, we wanted to investigate whether BIBF1120 could further affect the expression of downstream MAPK signaling pathways by inhibiting the VEGFR2, FGFR1, and PDGFR pathways.

To further study the role of BIBF1120 in DR, after BIBF1120 is injected into the vitreous cavity, streptozotocin (STZ) can increase the phosphorylation ratio of VEGFR2, FGFR1, PDGFR*β*, and MAPK signaling pathways in DR mice without damaging the retinal structure, function, or intraocular toxicity. VEGFR2, FGFR1, and PDGFR mRNA expression was examined by RT-PCR test. *In vitro* experiments showed that BIBF1120 and MAPK inhibitors inhibited the activation of p-JNK, p-p38, and p-ERK1/2 in the MAPK pathway of high glucose-treating human retinal microvascular endothelial cells (HRMECs) and further inhibited the proliferation, migration, and formation of tubes, but BIBF1120 had a better effect. These findings may help to clarify the mechanism and role of BIBF1120 in DR and suggest a protective therapeutic agent for DR patients.

## 2. Materials and Methods

### 2.1. Animals and Experimental Design

Adult male C57BL/6J mice (8 weeks old, 18.5–20.6 g, blood glucose level: 5.1–8.1 mmol/L) were bought from the Laboratory Animal Service Center of Nantong University. Animal research follows the Association for Research in Vision and Ophthalmology (ARVO) Statement on the Use of Animals in Ophthalmology and Vision Research. The Institutional Animal Care and Use Committee of Nantong University (S20210309-002) gave its approval to all animal experiments that strictly follow the National Institutes of Health guidelines and international ethical guidelines for the care and use of laboratory animals.

Animals were placed in a single standard cage and the environmental conditions were maintained at humidity (55% ± 2%), standard temperature (20 ± 2°C), and a 12-hour dark/light cycle, allowing free access to food and water. After overnight fasting, the mice were allocated to control nondiabetic mice group (Normal group, *n* = 25) and diabetic group (DR group, *n* = 75) at random. STZ (Sigma, USA; STZ solution was prepared in 0.1% mol/L citrate buffer, pH 4.5) were administrated intraperitoneally to mice in the DR group for 5 days at a dose of 60 mg/kg. An equal volume of STZ-free citrate buffer was employed for the normal group. On day 8, after the last injection, blood glucose was checked through the tail vein. In the experimental design, STZ mice were used as type 2 diabetic mice if blood glucose levels were over 16.7 mmol/L. The DR group was parted randomly into 2 groups (*N* = 30 in each group): DR group (intravitreal injection of 1 *μ*L sterile phosphate saline buffer (PBS, pH 7.4)) and BIBF group (DR mouse with intravitreal injection of 1 *μ*L BIBF1120 (concentration 200 nM) solution). The blood glucose and body weight of mice in each group before injection and the body weight and blood glucose at 2 months, 4 months, 6 months, and 8 months following injection were monitored and recorded. Mice were sacrificed by intraperitoneally injecting 1% sodium pentobarbital solution 24 weeks after injection. Mouse eyeballs were obtained and the samples were preserved at particular temperatures for upcoming imaging or biochemical analysis.

### 2.2. Intravitreal Injection

When STZ was induced in DR mice for 6 months, intravitreal injection was performed in the right eye of the mice. Under aseptic conditions, intravitreous injection was performed on the mice, followed by an intraperitoneal injection of 3% pentobarbital sodium for anesthesia, and compound tropicamide eye drops were utilized to dilate the pupils. Covering both eyes of the mouse with sodium hyaluronate, separating the eyelids of the mouse, pressing to make the eyeball protruding, piercing the eye with a 2 *μ*L microinjector vertically 0.5 mm behind the temporal corneal limbus, and then turning to the back to avoid damaging the lens. According to the previous study [24], the anesthetized mouse is a single shot received intravitreal injection of 1 *μ*L BIBF1120 (concentration 200 nM, Medchem Express Ltd. perfectly diluted in sterile-PBS for intravitreal injections) solution, and the equivalent amount of 0.1% DMSO slowly administered within 30 s, so that the liquid is fully diffused. Later, fundus evaluation showed whether there were any adverse reactions caused by intravitreal injection. Tobramycin eye drops were applied topically 3 times a day within one week after the intravitreal injection. Mice with retinal hemorrhage following injection were not included.

### 2.3. Assessment of Fasting and Weight

To verify the effect of STZ-induced DR, fasting plasma glucose was measured from the last STZ injection with the same glucometer (Bayer, Germany). We performed fasting plasma glucose measurements every two months by assessing blood glucose and weight, which were plotted by linking 4-time points in each group.

### 2.4. Terminal Deoxynucleotidyl Transferase dUTP Nick End Labeling (TUNEL) Analysis

The glass slides carrying 10 mm frozen sections of mouse eyes were dried for 30 minutes at ambient temperature. Both 0.1% sodium citrate and 0.1% triton X-100 were employed to permeabilize the tissues on ice for 2 min. With the use of an in situ cell death detection kit with TMR green (12156792910, Sigma-Aldrich), TUNEL labeling is carried out. The slices were covered with a paraffin film and incubated at 37°C for 60 minutes. Then, these parts were counterstained with DAPI. The cover glass is installed. The slices were observed and pictures were taken in a fluorescence microscope.

### 2.5. Hematoxylin-Eosin (HE) Staining of Retinal Sections

Fixation with 4% paraformaldehyde for 24 h, dehydration, and embedding in paraffin were performed in sequence on mouse eyeballs. For histopathological examination, tissues were cut into a thickness of 5 *μ*m and stained with eosin and hematoxylin. To remove paraffin, tissue sections were first baked at 60°C until xylene was visible, and then, they were immersed sequentially in a range of ethanol and ultrapure water concentrations. Subsequently, the slices were stained with hematoxylin solution and eosin in sequence, and sealed with neutral resin. Retinal pathological changes were identified and photographed by an optical microscope (Olympus, Tokyo, Japan).

### 2.6. Quantitative Reverse Transcription Polymerase Chain Reaction (qRT-PCR)

The total RNA from mouse retina on the 7^th^ and 14^th^ day after BIBF1120 treatment was separated using RNAiso Plus extraction kit (Takara, Japan). Reverse transcription to synthesize cDNA was processed as per the instructions of reverse transcription PCR kit (Takara, Japan), and the concentration and purity of cDNA were checked. QRT-PCR was executed with the use of a real-time PCR kit (Takara, Japan). Cycling conditions: 95°C for 30 s, 95°C for 40 s, 58°C for 40 s, and 72°C for 35 s, for a total of 40 standard PCR cycles; the gene expression values were normalized according to GAPDH mRNA and quantified using the 2^−ΔΔCt^ method.  Table 1 lists the primer sequences used.

### 2.7. Cell Culture and Treatment

HRMECs were offered by the American Type Culture Collection (ATCC CRL‐2299, Manassas, VA). HRMECs were grown in DMEM low-glycemic medium (5.5 mM glucose) containing 100 *μ*g/mL streptomycin, 100 U/mL penicillin, and 10% fetal bovine serum. Upon developing in a 5% CO_2_ and 37°C cell incubator, the medium was renewed every 3 days. HRMECs were stimulated with 25 mM (high sugar group) glucose for 24 h to construct an *in vitro* model of DR. According to different treatment conditions, the cells were divided into 7 groups, namely, normal group (NG, 5.5 mM glucose), high-mannitol group (HG, 25 mM mannitol), high-glucose group (HG, 25 mM glucose), high-glucose + DMSO group (HG + DMSO, 25 mM glucose + 20 *μ*M DMSO), high-glucose + BIBF1120 group (BIBF, 1 *μ*M), high-glucose + PD98059 (PD98059, 10 *μ*M, ERK inhibitor, Selleck.cn) group, high-glucose + SB600125 (SB600125, 10 *μ*M, JNK inhibitor, Selleck.cn) group, and high-glucose + SB203580 (SB203580, 10 *μ*M, p38 inhibitor, Selleck.cn) group.

### 2.8. Cell Counting Kit-8 (CCK-8) Assay

To continue the culture, 1 × 10^4^ cells per well were introduced into a 96-well plate and placed in an incubator. When the cells were fully adhered to and in good condition the next day, different concentrations of BIBF1120 were supplied to each well for 24 h pretreatment. Before 2 hour of incubation in a 37°C cell incubator, 100 *μ*L of fresh medium with the CCK8 reaction solution (10 *μ*L) was supplemented into each well. Finally, an automatic microplate reader was taken to detect the absorbance at 450 nm.

### 2.9. 5‐Ethynyl‐20‐Deoxyuridine Assay

HRMEC proliferation ability was evaluated by an EdU Kit (Cell Light EdU DNA Imaging Kit, RiboBio) as per the manufacturer's instructions. Images were obtained and observed under a microscope (Tokyo, Japan). Cell proliferation activity was assessed by the ratio of 5‐ethynyl‐20‐deoxyuridine (EdU)‐stained cells (red fluorescence) to DAPI‐stained cells (blue fluorescence).

### 2.10. Cell Migration Assay

If the cells reached over 90% confluence, a pipette tip (10 *μ*L) was chosen to scratch the cell monolayer. The cells were subsequently grown in a serum-free medium with or without VEGFR and MAPK pathway inhibitor for 12 h. After scratching, images were obtained at 0 and 12 h. The results came from the measured areas in 6 fields selected at random in a well, with six repetition of independent pooled cell samples. These finished the scratch assay. For the Transwell migration assay, HRMECs (2.5 × 10^5^) were suspended in a serum-free DMEM (250 *μ*L) and inoculated into the upper chambers of Transwell plates (24 wells, Corning Inc., Corning, NY). DMEM (600 *μ*L) containing 10% FBS was introduced into the lower chambers. Twenty-four hours later, 0.1% crystal violet and methanol were adopted for the staining of the bottom of each chamber insert. An Olympus IX70 inverted microscope (Tokyo, Japan) was used to image and count the cells, ImageJ software (NIH, Bethesda, MD) to calculate the mean cell count of four stained membrane images. Each assay was carried out three times.

### 2.11. Tube Formation Assay

Using an *in vitro* Matrigel‐based tube formation assay, HRMEC angiogenic ability was evaluated. Briefly, the plate (96-wells) was precoated with Matrigel basement membrane matrix (50 *μ*L) and then incubated for 30 min at 37°C. HRMECs (3 × 10^4^) were inoculated on Matrigel, and they received the recommended treatment for 6 hours. The images were obtained from five microscopic fields selected at random. An IX70 microscope was chosen to examine the cumulative tube length at a magnification of ×200.

### 2.12. Western Blot Analysis

The proteins were isolated from HRMECs using a Tissue Lyser II or retinal tissues homogenized in different groups, and Bradford assay was performed to determine its concentration. Sample buffer containing 20% of 2‐mercaptoethanol was applied to dilute the protein (5 mg/sample). The samples underwent 10% sodium dodecyl sulphate-polyacrylamide gel electrophoresis (SDS‐PAGE) after being heated for 10 min at 95°C. Subsequent to electroblot the separated proteins onto a polyvinylidene fluoride (PVDF) membrane, the PVDF membrane was subjected to blocking with 50 mg/mL skimmed milk in PBS at ambient temperature for an hour. Membranes were developed with primary antibodies anti-p-FGFR1 (60325-1-Ig; Proteintech), anti-FGFR1 (A0082; ABclone), anti-VEGFR2 (AB52917; Abcam), anti-p-VEGFR2 (AP0382; ABclone), anti-p-PDGFR (AP1062; ABclone), anti-PDFGR (17. A2180; ABclone), anti-ERK1/2 (#9102s; Proteintech), anti-p-ERK1/2 (#4370s; Proteintech), anti-p38 (#9212s; Proteintech), anti-p-p38 (#9211L; Proteintech), anti-JNK(#9252s; Proteintech), anti-p-JNK(9251L; Proteintech), and anti-GAPDH (sc‐365062; Santa Cruz) overnight at 4°C. After that, the membrane was treated for 2 hour at ambient temperature with secondary antibodies goat anti-mouse (ab6789; Abcam) or goat anti-rabbit (ab6721; Abcam). An ECL chemiluminescence system (Beyotime Institute of Biotechnology) was adopted to detect immunoreactive proteins, a chemiluminescence gel imager (Bio‐Rad) to capture their images, and ImageJ software to measure the band intensities.

### 2.13. Statistical Analyses

Statistics were conducted with SPSS 19.0 and GraphPad Prism 7.0 software. The two-group comparison was evaluated by the Student *t*-test. Using one-way ANOVA, comparisons between multiple groups were carried out. A value of *P* < 0.05 was deemed statistically significant. All results were presented as the mean ± standard deviation (SD). At least three rounds of each experiment were completed.

## 3. Results

### 3.1. General Characteristics of Diabetic Mice

**Figure 1:**
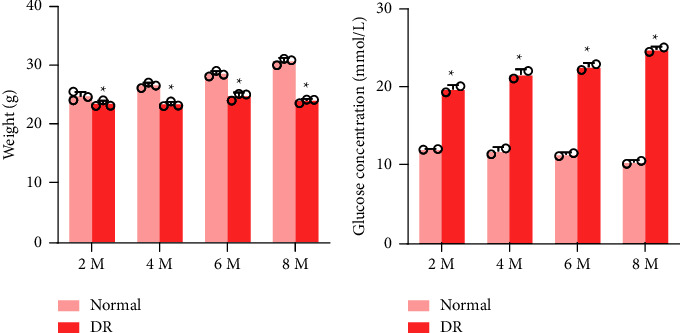
General characteristics of diabetic mice. (a) The weight of the DR group mice and normal group. (b) Blood glucose levels of mice in the DR group and normal group ^*∗*^*P* < 0.05 vs. normal group.

In order to build a DR mouse model, this study induced DR mice by intraperitoneal injection of STZ. The findings were that, the body weight of the DR mice did not increase over time but less than the normal group (*P* < 0.05) (Figure 1(a)), and the blood glucose level elevated obviously (*P* < 0.05) (Figure 1(b)). These indicated that, DR mice were successfully obtained.

### 3.2. BIBF1120 Ameliorated STZ-Induced DR

**Figure 2:**
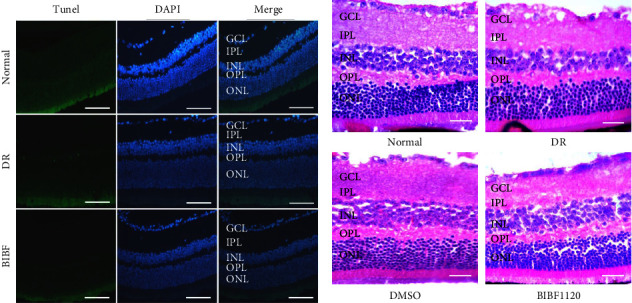
BIBF1120 ameliorated STZ-induced DR. (a) TUNEL (green) and DAPI (blue) immunofluorescence staining of retinal cryosections from the normal, DR, and DR plus BIBF1120 groups, scale bar = 50 *μ*m. (b) Mouse retina complexes stained with H&E in the normal, DR, and DR + BIBF1120 groups and DR + DMSO groups, scale bar = 100 *μ*m. GCL: ganglion cell layer; IPL: inner plexiform layer; INL: inner nuclear layer; IS: inner segment; OPL: outer plexiform layer; ONL: outer nuclear layer; OS: outer segment.

To further study the treatment and possible adverse reaction of BIBF1120 on DR mice, TUNEL staining was performed on retinal cryosections. The findings included that the retinal cell apoptosis level in the DR group was much higher than that in the normal group, and relative to the DR group, retinal cell apoptosis level was significantly reduced following the BIBF1120 treatment (Figure 2(a)). In terms of H&E staining results, the retinal ganglion cells, with smooth retinal surface and complete structure, in each layer of the mice in the normal group were neatly arranged. In the DR group, the inner nuclear layer of the retina became thinner, and retinal ganglion cells were discretely distributed. Compared with the DR group, BIBF1120 reduced retinal histopathological changes in STZ-induced DR mice (Figure 2(b)). These indicated that BIBF1120 treatment could improve the STZ-induced DR.

### 3.3. BIBF1120 Can Reduce the Expression of Retinal Angiogenesis-Related Molecules in DR Mice

**Figure 3:**
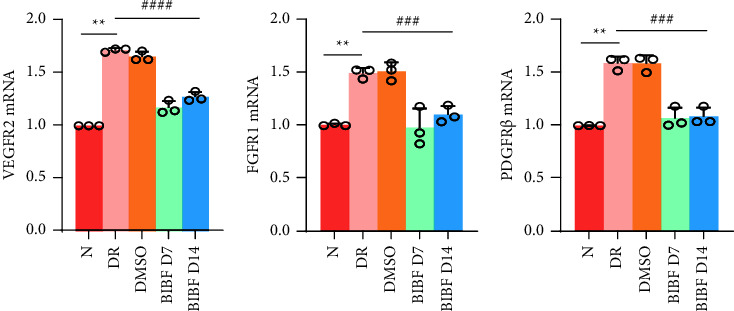
BIBF1120 can reduce the mRNA expression of retinal angiogenesis-related molecules in DR mice. (a–c) Analysis of VEGFR2 (a), FGFR1 (b), and PDGFR*β* (c) mRNA in the mouse retinas on the 7^th^ and 14^th^ days after intravitreal injection of BIBF1120. ^*∗∗*^*P* < 0.01 vs. normal group, ^###^*P* < 0.001 vs. DR group.

To assess the impact of BIBF1120 on the angiogenesis-related molecule expression in the retina of DR mice, this study utilized qRT-PCR to examine VEGFR2, FGFR1, and PDGFR*β* mRNA expression levels in the retina on the 7^th^ and 14^th^ day after BIBF1120 treatment. The histogram displayed that VEGFR2, FGFR1, and PDGFR*β* mRNA expression levels in the DR group increased markedly relative to those in the normal group (*P* < 0.01) (Figures 3(a)–3(c)). In contrast to the DR group, the VEGFR2, FGFR1, and PDGFR*β* expression levels were significantly reduced on the 7^th^ and 14^th^ day after BIBF1120 treatment (*P* < 0.001) (Figure 3).

### 3.4. BIBF1120 Inhibits the Expression of Angiogenesis-Related Proteins and the MAPK Signaling Pathway in the Retinas of DR Mice

**Figure 4:**
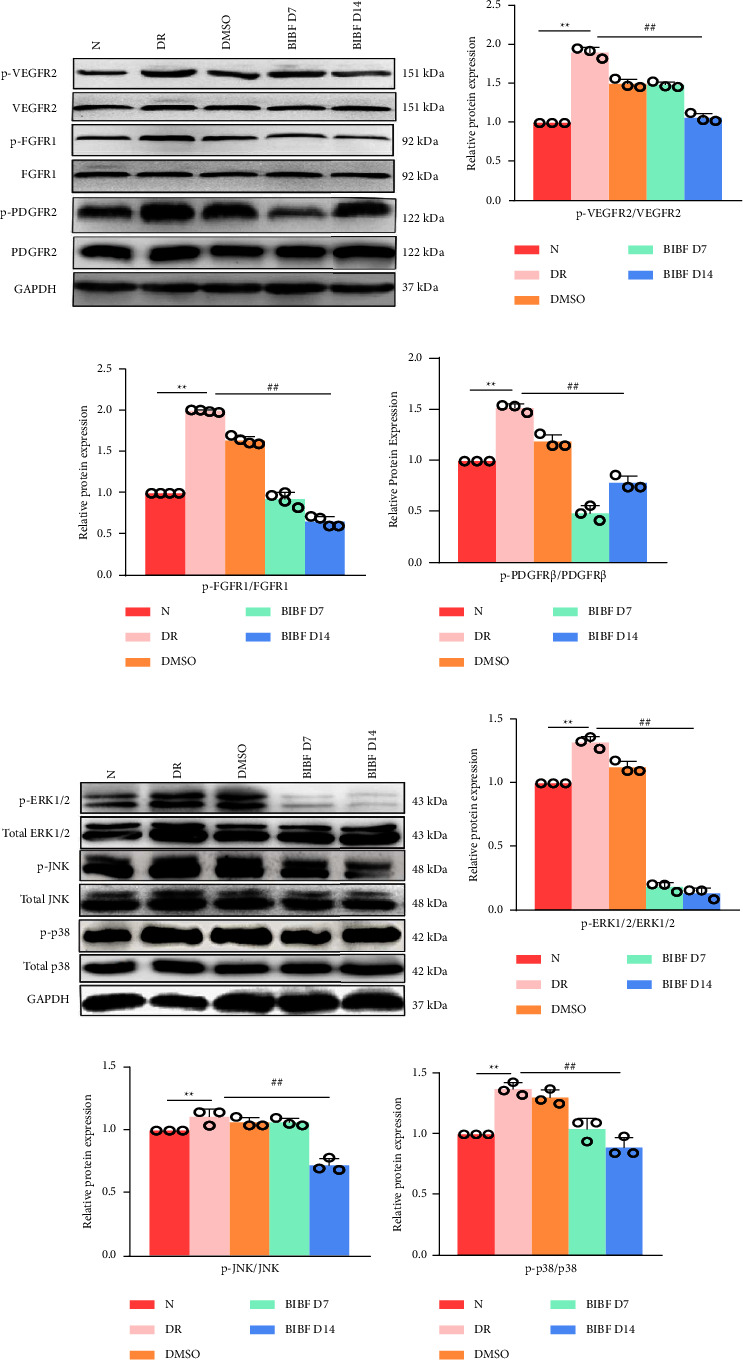
BIBF1120 inhibits the expression of angiogenesis-related proteins and the MAPK signaling pathway in the retinas of DR mice. (a) Angiogenesis-related proteins in the retina of mice in each group detected by Western blots; (b–d) quantitative analysis of p-VEGFR2/VEGFR2 (b), p-FGFR1/FGFR1 (c), and p-PDGFR*β*/PDGFR*β* (d); (e) MAPK signaling pathway related proteins in the retina of mice in each group detected by Western blots; (f–h) quantitative analysis of p-ERK1/2/ERK1/2 (f), p-JNK/JNK (g), and p-p38/p38 (h). ^*∗∗*^*P* < 0.01 vs. normal group, ^##^*P* < 0.01 vs. DR group.

Subsequently, we further examined the expression of angiogenesis-related proteins and MAPK signaling pathways in the retina to investigate the therapeutic mechanism of BIBF1120 in DR mice. Western blot analysis revealed that relative to the normal group, the p-VEGFR2, p-FGFR1 and p-PDGFR*β* as well p-p38, p-JNK and as p-ERK1/2 protein expressions in the DR group were obviously up-regulated (*P* < 0.01). On the 7^th^ and 14^th^ days after BIBF1120 treatment, those protein expressions were notably down-regulated (*P* < 0.01) (Figures 4(a)–4(h)). It indicated that BIBF1120 may treat DR by inhibiting angiogenesis-related proteins and MAPK signaling pathways in the retina.

### 3.5. The Effect of Intervention under Different In Vitro Conditions on the Vitality of HRMECs

**Figure 5:**
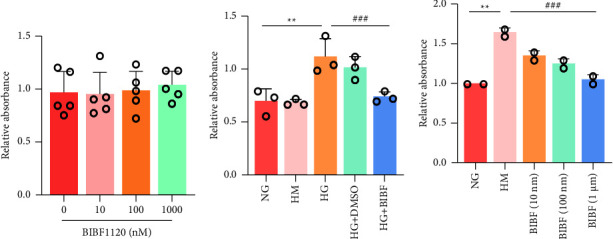
The effect of BIBF1120 on the viability of high glucose-induced HRMECs: (a) the effect of different concentrations of BIBF1120 on cell viability; (b) the effect on cell viability under hypertonic conditions; (c) the effect different concentrations of BIBF1120 treatment on cell viability under hypertonic conditions. (^*∗∗*^*P* < 0.01 vs. control group; ^###^*P* < 0.001 vs. high-glycemic group).

The cell viability was evaluated by CCK-8. BIBF1120 at each concentration had no significant effect on cell viability under normal sugar concentrations (Figure 5(a)). In comparison with the normal group, the HM group exerted no obvious effect on the cell viability, so the effect of hypertonicity on the cells was excluded. Under high glucose stimulation for 24 h, cell viability increased (*P* < 0.01), while under different concentrations of BIBF1120 treatment, cell viability decreased (*P* < 0.01), especially 1 *µ*M BIBF1120. (Figures 5(b) and 5(c)). Therefore, 1 *µ*M BIBF1120 was selected to stimulate HRMECs for 24 h to test its effect on the biological function of HRMECs.

### 3.6. BIBF1120 Suppresses the Tube Formation, Migration, and Proliferation of HRMECs In Vitro

**Figure 6:**
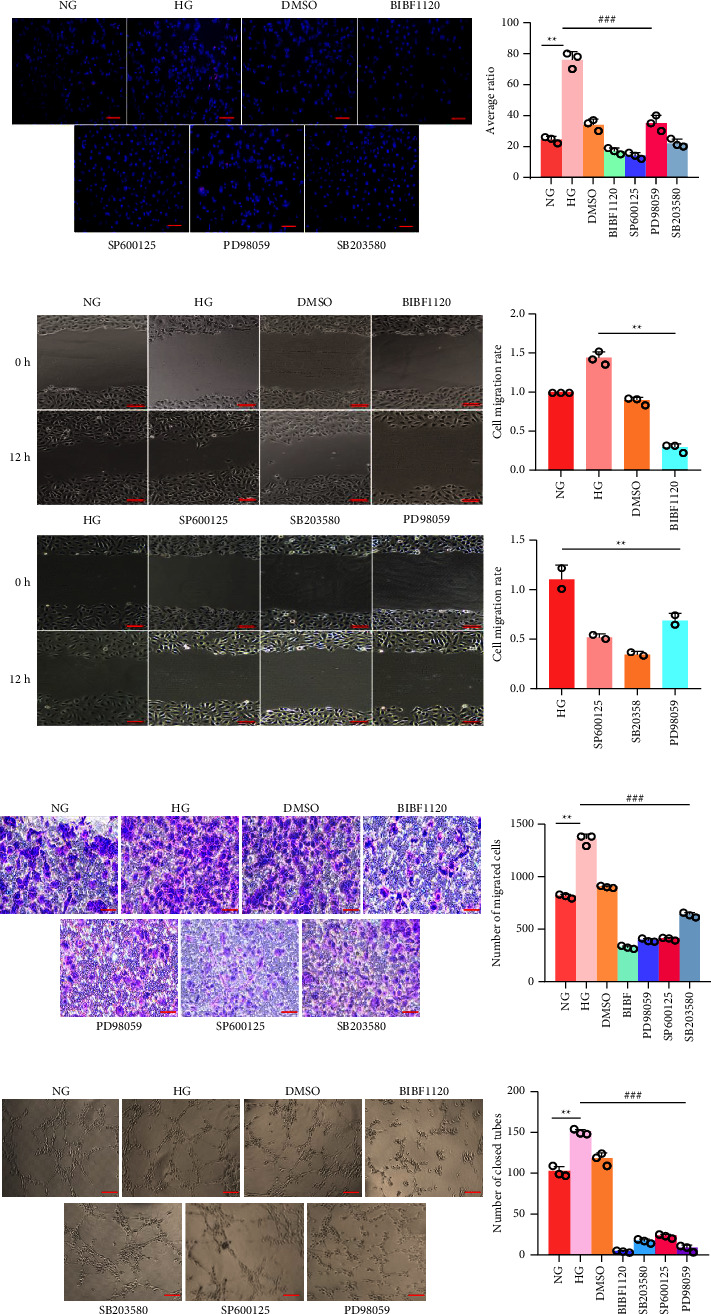
BIBF1120 suppresses the tube formation, migration, and proliferation of HRMECs *in vitro*. (a) The proliferation capability of HRMECs measured by an EdU assay. The EdU‐positive cell ratio was analyzed (red: proliferating cells, blue (DAPI): nucleus, scale = 200 *μ*m). (b) Scratch test to detect the migration of HRMECs (scale bar = 20 *μ*m). (c) A the migration capability of HRMECs detected by a transwell assay (scale bar = 20 *μ*m). The number of migrated cells was counted. (d) The number of closed tubes obtained by a tube formation assay (scale bar = 100 *μ*m). ^*∗∗*^*P* < 0.01 vs. the normal group; ^###^*P* < 0.001 vs. high glucose group. DMSO: dimethyl sulfoxide; EdU: 5‐ethynyl‐20‐deoxyuridine.

With the aim of further identifying the antiangiogenic effects of BIBF1120 in HRMECs under high glucose stimulation, BIBF1120 and MAPK pathway inhibitor were added to HRMECs under high glucose conditions. BIBF1120 and MAPK inhibitor under high glucose conditions inhibited the proportion of EdU‐positive cells in the high glucose group relative to the normal group (*P* < 0.01). In comparison with the high glucose group, both inhibited the proportion of EdU‐positive cells (*P* < 0.01), but BIBF1120 had the most significant inhibitory effect (Figure 6(a)). BIBF1120 affected the migration of high glucose-induced HRMECs. In comparison to the high glucose group, BIBF1120 reduced the proportion of the HRMEC migration area and the number of migrating cells (*P* < 0.01); after adding BIBF1120 and MAPK pathway inhibitors to the high-glucose medium for 24 h, the proportion of the HRMEC migration area and the number of migrating cells mounted in the high-glucose group relative to the normal group (*P* < 0.01); when the inhibitor was added, the proportion of cell migration areas and the number of migrating cells had different degrees of inhibition (*P* < 0.01), but BIBF1120 inhibited the greatest effect (Figures 6(b) and 6(c)). BIBF1120 affected the *in vitro* tubes of formation of HRMECs treated with high glucose. The high glucose group had a larger number of closed tubes than the normal group (*P* < 0.01), and the number of closed tubes was inhibited to different degrees with the addition of inhibitors in the high glucose group (*P* < 0.01) (Figure 6(d)). However, BIBF1120 inhibits the most. Therefore, BIBF1120 suppressed the tube formation, migration, and proliferation of HRMECs *in vitro*.

### 3.7. The Effect of BIBF1120 and Different Inhibitors on the MAPK Signaling Pathway in HRMECs Induced by High Glucose

**Figure 7:**
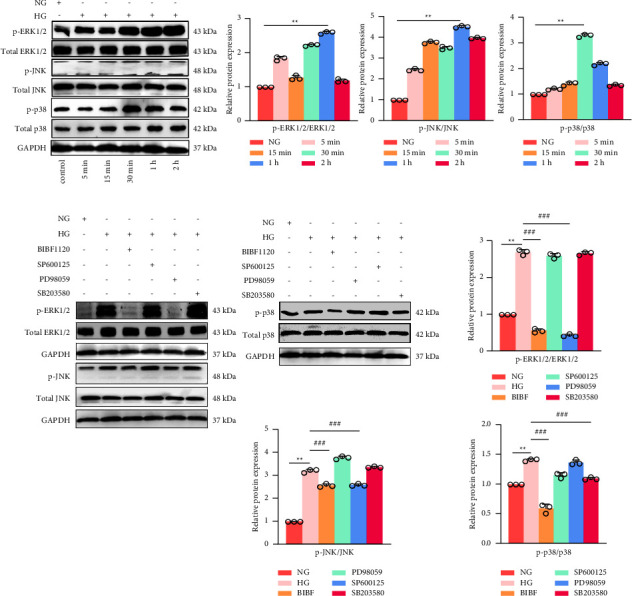
The effect of BIBF1120 and Different Inhibitors on the expression MAPK/ERK1/2/JNK/p38 signaling pathway of HRMECs with high glucose. (a)-(b) Western blot results of p-JNK/total JNK, p-p38/total p38 and p-ERK/total ERK. ^*∗∗*^*P* < 0.01 vs. normal group. ^###^*P* < 0.001 vs. high glucose group.

Whether the MAPK signaling pathway participates in the signal transduction of HRMECs induced by high glucose is determined by Western blot detection. HRMECs were stimulated for 5 min, 15 min, 30 min, 1 h, and 2 h under high glucose conditions. The high glucose group presented higher p-JNK/total JNK, p-p38/total p38, and P-ERK/total ERK ratios than those of the normal group (*P* < 0.01). The p-ERK1/2 and p-JNK expression was highest at 1 h, while p-p38 was highest at 30 min (Figure 7(a)). After the addition of BIBF1120, PD98059, SP600125, and SB203580 inhibitors under high glucose conditions for 1 h, only BIBF1120 and PD98059 inhibited p-ERK1/2 and p-JNK expression, which have similar degrees of inhibition (*P* < 0.01) (Figure 7(b)); while the p-p38 expression was only inhibited by the BIBF1120 and SB203580 inhibitors (*P* < 0.01), and BIBF1120 has a greater degree of inhibition (*P* < 0.01) (Figure 7(b)). According to these results, BIBF1120 inhibits the MAPK/ERK1/2, JNK, and p38 signaling pathways induced by 1 h of high glucose.

## 4. Discussion

DR is one of the common complications of diabetes, and as the incidence of diabetes mellitus rises, so does its incidence. In recent years, with changes in people's lifestyles, increases in work pressures, and changes in the environment, the incidence of diabetes has presented an obvious rising trend, which has caused an obvious increase in the incidence of diabetic retinopathy [1]. Retinal neovascularization and the emergence of PDR are the results of the pathological changes include loss of retinal perivascular cells and increase in proliferation and migration of HRMECs [25]. The cascade reaction of VEGF as a promoter to activate VEGFR2 is the core signaling pathway in the process of angiogenesis, which can encourage the survival, migration, and proliferation of vascular endothelial cells [5]. Anti-VEGF medication is regarded as a viable DR treatment [26–29]. This study demonstrated the function and related mechanism of a potential anti-VEGF drug BIBF1120 in protecting and treating DR.

BIBF1120 is a small molecule tyrosine kinase inhibitor, acts on the 3 receptor families (VEGFR2, FGFR1, PDGFR*β*) associated with angiogenesis [30]. RTKs is a cell surface growth factor receptor that exhibits intrinsic ligand-controlled tyrosine kinase activity [31], closely related to the occurence of ocular neovascular diseases [32]. RTK inhibition is a useful alternative treatment, according to several research [33–35]. Recently, the combination of inhibitors against different RTK ligand (especially VEGFR and PDGFR) ligands seems very promising in the clinic. In a xenograft tumor mouse model, BIBF1120 showed preclinical antiangiogenic activity and decreased tumor microvessel density [30]. In the rat oxygen-induced retinopathy (OIR) model, pathological retinal neovascularization was eliminated, and normal blood vessel formation was enhanced. Oral BIBF1120 can effectively treat pathological neovascularization, is well tolerated in animals, and tends to reduce the avascular area. It is an effective inhibitor of neovascularization-associated ischemic oxidative retinopathy [24]. In this study, BIBF1120 reduced the mRNA expression and STZ-induced phosphorylation of VEGFR2, FGFR1, and PDGFR*β* in the retinas of diabetic mice.

Additionally, BIBF1120 can weaken the migration, differentiation and proliferation of fibroblasts [36]. Smooth muscle cells, pericytes and endothelial cells are among the vascular cell types in the lung that BIBF1120 prevents from proliferating [37]. Human pulmonary artery smooth muscle cells can be prevented from proliferating by BIBF1120 when they are stimulated by PDGF under basal circumstances [38]. In an animal model of bleomycin-induced pulmonary fibrosis, BIBF1120 showed anti-fibrosis, anti-inflammatory and vascular remodeling activities, normalized the twisted vascular structure and inhibited alveolar macrophage and alveolar epithelium type II cell proliferation [39–41]. In this study, among the HRMECs induced by high glucose *in vitro*, BIBF1120 was found to suppress the lumen formation, migration and proliferation of HRMECs.

The MAPK signaling pathway is an important signal transduction pathway that includes p38 MAPK, JNK, and ERK, which are believed to participate in several pathological and physiological reactions of the human body. A signal transduction system called mitogen-activated protein kinase (MAPK) is implicated in a number of physiological activities, like survival, death, differentiation, proliferation, and gene expression of numerous cells [42]. The MAPK signaling pathway, which contributes to the occurrence of DR, has been demonstrated to be crucial in a number of pathogenic pathways and pathological angiogenesis, such as controlling cell proliferation and cycle entry [15]. Our study found that BIBF1120 can reduce the MAPK (p-JNK, p-ERK1/2, p-38) expression in HRMECs induced by high glucose *in vitro* and further inhibit the lumen formation, migration, and proliferation ability of HRMECs *in vitro*.

## 5. Conclusions

In summary, this study is the first to examine the protective effect of BIBF1120 on DR. BIBF1120 plays a protective role in STZ-induced DR and high glucose-induced HRMECs by inhibiting the VEGFR2, FGFR1, PDGFR*β*, and MAPK signaling pathways, suggesting that targeting this pathway may be a successful approach for treating DR. Further research is required to be conducted to fully understand the protective effect of BIBF1120 on DR, albeit this study offers scientific and experimental evidence for the possibility of clinically employing BIBF1120 as a therapeutic drug in DR treatment.

## Figures and Tables

**Table 1:** Primer sequence.

Genes	Primer sequence
VEGFR2	Forward: 5′-GCAATGATGAAGCCCTGGAGT-3′
	Reverse: 5′-CTGAACAAGGCTCACAGTGAT-3′

FGFR1	Forward: 5′-TGGAGTTAATACCACCGACAAA-3′
	Reverse: 5′-GATGATGATCTCCAGGTACAGG-3′

PDGFR*β*	Forward: 5′-GTCAAGATGCTGAAATCGACAG-3′
	Reverse: 5′-GGGGTCCAAGATGACTCATAAT-3′

GAPDH	Forward: 5′-TCGTACCTTTCTCACCACAGTATCTAG-3′
	Reverse: 5′-GAAAACTAAGACACCTCCCCATCATC-3′

## Data Availability

The data used to support the findings of this study are available from the corresponding author upon request.
